# Color Characteristics of High Yttrium Oxide–Doped Monochrome and Multilayer Partially Stabilized Zirconia upon Different Sintering Parameters

**DOI:** 10.1055/s-0044-1787700

**Published:** 2024-06-28

**Authors:** Niwut Juntavee, Apa Juntavee, Chutikarn Jaralpong

**Affiliations:** 1 Department of Prosthodontics, Faculty of Dentistry, Khon Kaen University, Khon Kaen, Thailand; 2 Department of Preventive Dentistry, Division of Pediatric Dentistry, Faculty of Dentistry, Khon Kaen University, Khon Kaen, Thailand; 3 Division of Biomaterials and Prosthodontics Research, Faculty of Dentistry, Khon Kaen University, Khon Kaen, Thailand

**Keywords:** color appearance, contrast, sintering, temperature, time, translucency, opalescence

## Abstract

**Objectives** Sintering influences the optical properties of zirconia. This study examined the effect of altering sintering temperature and time of monochrome (Mo) and multilayer (Mu) 5 mol% yttria-partially stabilized zirconia (5Y-PSZ) on color characteristics.

**Materials and Methods** Three hundred specimens (width × length × thickness = 10 × 20 × 2 mm) were prepared from Mo and Mu (with cervical [C], middle [M], and incisal [I] region) 5Y-PSZ and randomly sintered at decreasing (Td: 1,450°C), regular (Tr: 1,500°C), and increasing (TI: 1,550°C) sintering temperature, with extremely short (He: 10 minutes), ultrashort (Hu: 15 minutes), short (Hs: 30 minutes), and regular (Hr: 135 minutes) sintering time ( *n* = 15/group). Color appearance ( *E _W_* ), translucency parameter (TP), contrast ratio (CR), opalescence parameter (OP), and color appearance difference (∆ *E*_diff_ ) were evaluated in the CIE L*a*b* system. Microstructures were evaluated by scanning electron microscope (SEM) and X-ray diffractometer (XRD).

**Statistical Analysis** Analysis of variance (ANOVA) and Bonferroni comparisons were determined for significant differences ( *p* < 0.05).

**Results** Significant differences in color parameters upon zirconia type, sintering temperature, and sintering time, and their interactions were indicated ( *p* < 0.05). Increasing sintering temperature and extended sintered time resulted in larger grain, reduced tetragonal-to-monoclinic phase transformation, and significantly increased the TP and OP, but decreased the CR and ∆ *E*_diff_ ( *p* < 0.05). Decreasing sintering temperature and time led to clinically unacceptable color appearance.

**Conclusion** Mo was found to be more translucent than Mu. To achieve the most favorable optical properties, increasing sintering temperature and extending sintering time are recommended. Decreasing sintering temperature is not suggested. However, shortened sintering time is feasible, but it needs sintering with increasing sintering temperature to achieve a promising color appearance.

## Introduction

The progression of digital dentistry has been increasingly using a diversity of ceramic materials in restorative dentistry due to their ability to simulate natural tooth color, biocompatibility, and resistance to fracture. 1 Still, the ability to mask undesirable color from the underneath structure and fracture resistance are limited for extensive ceramic reconstructions. 2 Several ceramic materials have been improved in aesthetic requirement as well as the durability to function as the long-span fixed prosthesis. Zirconia is considered an attractive ceramic owing to its proximity to the color of the human natural tooth, remarkable fracture resistance, high corrosion resistance, minute thermal conductivity, and relatively stable dimension. 3 Zirconia has been progressively used in restorative dentistry as a substructure for ceramic veneering restoration, partial or full coverage restoration, and full arch fixed prostheses. It naturally occurs in the polymorphic crystalline structures: monoclinic (m), tetragonal (t), and cubic (c) phases. The monoclinic crystal structure appears at normal room temperature (RT), then converts to the tetragonal crystal structure at 1,170°C. Upon reaching 2,370°C, the change from the tetragonal to cubic crystal structure happens, and stays in the cubic phase until reaching the melting point at 2,680°C. 4 The t and c phases of zirconia become stable at RT via the integration of some dopants, for instance, yttrium oxide (Y _2_ O _3_ ), or cerium oxide (CeO _2_ ). Yttrium oxide (Y _2_ O _3_ ) at 3 mol% has been primarily used as an oxide stabilizer of zirconia in dentistry for developing yttria-stabilized tetragonal zirconia polycrystals (Y-TZP) and holds notable strength above other dental ceramics. 5 Upon being stimulated with stress, heat, or humidity, the phase of 3 mol% yttria-partially stabilized zirconia (3Y-TZP) could shift from the t to m phase, causing 4 to 5% microstructural enlargement. 6 The volumetric alteration provokes compressive stress to resist crack propagation, recognized as “transformation toughening,” which provides peculiar strength for zirconia. 7 The 3Y-TZP is fairly opaque because it comprises entirely distinctive crystalline structures with dissimilar refractive indices, causing extreme light scattering and dispersed reflection more than glass-based ceramics. 2 It was principally used as a substructure to support veneering porcelain to achieve a durable and aesthetic translucency restoration. However, the occurrences of porcelain delamination and chipping were broadly described. 8 9 This consequently steers the launch of monolithic 3Y-TZP to fabricate a stronger restoration at a reduced thickness of 0.5 to 0.75 mm, in comparison to ceramic veneered zirconia and other ceramic materials. 10 11 Yet, the traditional first-generation monolithic 3Y-TZP possessed minimal translucence. The rising sintering temperature of the traditional monolithic zirconia was capable of inducing a larger grain size, permitting better light transmission, hence pretty enriching translucence, but then diminishing the strength. 1 12 13 14 15 The following second-generation monolithic translucence zirconia was improved by reducing the quantity and particle size of aluminum oxide (Al _2_ O _3_ ). Once sintering, the Al _2_ O _3_ particles were reorganized and colonized along the grain boundaries. This enabled the reduction of light scattering from Al _2_ O _3_ particles and promoted more transmission of light throughout the zirconia grain, thus providing more translucence, nevertheless less translucence than glass-based ceramics. Lately, 5 mol% yttria-partially stabilized zirconia (5Y-PSZ) was commenced, which comprised 40 to 50% of c phase and reduced other phases to a degree of high-translucence zirconia. 16 17 The c phase employed a larger volume and developed extreme isotropic structure that presented a lesser amount of light scattered effect at the grain boundary, thus providing further translucency. Contemporary 5Y-PSZ was launched in a layered configuration that imitated the natural tooth color scheme from the cervical to incisal zones, whereas the monochrome pattern comprised solitary mono shade. 11

Color characteristics, comprising color perception, translucence, contrast, and opalescence, are the prime concerns in the selection of ceramics for aesthetic restoration. 18 Regarding color perception, the color appearance difference (∆ *E*_diff_ ) is used to determine the degree of perception of color difference. The amount of ∆ *E*_diff_ < 3 signified “clinically indifferent,” ∆ *E*_diff_ = 3–5 signified “clinically acceptable,” and ∆ *E*_diff_ > 5 signified “clinically unacceptable” appearance. 19 Translucence was defined through the amount of optical transmission throughout the material. This circumstance exists in between the opaqueness and transparency of the material. The more the optical transmission, the superior the translucence. 20 The optical characteristics are considered in terms of translucency parameter (TP), contrast ratio (CR), and opalescence parameter (OP). 15 The superior translucence material would exhibit a greater TP but lower CR value because these parameters are adversely correlated. 10 The dimensions of grains, crystalline microstructures, coloring pigments, and porosities were stated to influence the light path. 15 The restoration would look blueish once light is redirected from it and display an orange illusion as light transmits throughout. This occurrence is recognized as opalescence, which creates the restoration closely imitating the lifelike human enamel that has OP in the range of 19.8 to 27.6. 21

Several attempts have been made to improve the color characteristics of zirconia restoration through alteration of the sintered parameters, variation in the alumina composition, and change in the quantity of Y _2_ O _3_ . 10 A decrease in sintering temperature and sintering time exaggerated crystalline progression, density of the material, and pore size. 4 18 22 Increasing the sintering temperature enhanced translucency of monolithic zirconia. 1 However, increasing the sintering temperature beyond 1,600°C is not suggested owing to the decrease in flexure strength. 10 Lengthening the sintering time of 3Y-TZP notably progresses optical parameters through amplification of grain and ultimately initiates the t to m phase transformation. 18 Speedy cooling of 3Y-TZP expedites the greater grain size, together with shifting of t to m phase rendering better translucence while decreasing flexure strength. 4 22 The procedure in lessening plus relocation of alumina particles to the grain boundary enabled enhancing of translucence as well as strength. 16 Increasing Y _2_ O _3_ to approximately 5 mol% to become 5Y-PSZ increases the c phase by up to 40 to 50%. 3 The cubic configurations occupy a larger volume, and then produce a further isotropic structure to lessen the scattering of light at grain boundaries, and consistently allow light to pass through, thus causing superior translucence zirconia. 16 Nevertheless, a high concentration of Y _2_ O _3_ demonstrates less capability of transformation toughening, thus sacrificing flexure strength and toughness. 3 11

Fabrication of the zirconia restorations is generally performed via a milling process using a partial sinter blank and then sintered at 1,400°C for almost 7 to 12 hours to obtain a mature zirconia, which is a time-consuming process, and usually needs the designed restoration around 20 to 25% to compensate for shrinkage upon sintering. 14 Most clinicians prefer rapid sintering to obtain efficient production at cost-effectiveness. 10 Sintering parameters are commonly adjusted to achieve better restoration. Currently, multilayer monolithic zirconia has been produced with the incorporation of some pigment added among layers to imitate the gradually colored alteration of human teeth. 6 Likewise, several reports indicated the impact of coloring pigmentation on microstructure, color parameters, 6 20 23 and strength. 3 11 24 25

Sintering parameters are commonly adjusted to achieve better color characteristics of the restoration. Preceding studies examined the influence of heating speed, sintered time, and temperature on flexure strength and translucency of monochrome monolithic zirconia. 16 18 26 27 28 However, there is a shortage of studies on the impact of modifying sintered parameters on Mo and Mu 5Y-PSZ. Hence, this study intended to assess the color characteristics of Mo and Mu 5Y-PSZ once altering sintering temperatures and times. It was hypothesized that changing the sintering temperature and time would not affect the color characteristics of Mo and Mu 5Y-PSZ.

## Materials and Methods

This *in vitro* study was carried out based on the estimated sample size from the statistical data in Sailer et al 9 using G*power 3.1 software (Heinrich-Heine-Universität, Düsseldorf, Germany) with 90% power of the test, and *α* error = 0.05 as shown in equation (1) , which resulted in 15 samples per group.



where *Z _α_* = standard normal deviation = 1.96 ( *α* error = 0.05), *Z _β_* = standard normal deviation = 1.28 ( *β* error = 0.1), *µ*_1_ – *µ*_2_ = mean difference between groups = 0.8, and *s* = standard deviation ( *s*_1_ = 2.3, *s*_2_ = 1.5).

### Preparation Zirconia Specimens

Three hundred 5Y-PSZ specimens were initially prepared from presintered monochrome (Mo: Cercon xt, Dentsply Sirona, Charlotte, NC, United States) and multilayer (Mu: Cercon xt ML, Dentsply Sirona) blocks, shade A2 as shown in Table 1 . The diamond-impregnated disk (Mecatome T180, Presi, Eybens, France) was utilized in preparing specimens at a bigger dimension (width, length, and thickness of 12.5, 25, 2.5 mm, respectively) to compensate for contraction upon sintering, then ground flat with SiC abrasive (grit no. 1200) and afterward smoothed with 1-µm diamond suspended liquid in a polishing machine (Ecomet 3, Buehler, Lake Bluff, IL, United States). The specimens were ultrasonically cleaned for 10 minutes in a cleansing apparatus (Vitasonic II, Vita Zahnfabrik, Bad Säckingen, Germany) and finally dried up for 60 minutes at RT. Each zirconia sample was unintentionally assigned to 10 groups ( *n* = 15) according to the difference in sintering times (regular [Hr: 135 minutes], short [Hs: 30 minutes], ultrashort [Hu: 15 minutes], and extremely short [He: 10 minutes]), and the difference in sintering temperatures (regular [Tr: 1,500°C], decreasing [Td: 1,450°C], and increasing [Ti: 1,550°C]). The manufacturer sintering parameter at Hr and Tr was used as a reference. The process of sintering was accomplished in a furnace (inFire HTC, Dentsply Sirona) upon the heating rate of 22°C/min till 880°C and further heating at 11°C/min till reaching the final temperature, and then cooled down to RT at a rate of 35°C/min. The mature samples (width, length, and thickness of 10, 20, and 2 mm, respectively) were kept for 24 hours at RT before testing.

**Table 1:** (A) Material, batch number, and composition (wt%) of monochrome (Mo) and incisal (I), middle (M), and cervical layer (C) of multilayer (MU) 5 mol% yttria-partially stabilized zirconia (5Y-PSZ) used in this study and (B) list of word/phrase abbreviations (Abv.)

**A. Material, batch number, and composition (wt%) of 5Y-PSZ**													
**Material**	**Batch no.**	**Layer**	**Composition (wt%)**										
			**Zr**	**Y**	**O**	**Hf**	**Al**	**Si**	**Fe**	**Ca**	**Na**	**K**	**Other**
Cercon xt	18040682	Mo	74.86	7.57	15.01	2.24	0.15	0.02	0.12	0.03	0	0	0
Cercon xt ML	18041981, 18042302	MuI	72.24	7.50	18.00	1.88	0.18	0.02	0.09	0.03	0.04	0.01	0.01
		MuM	73.45	7.54	16.71	1.94	0.18	0.04	0.07	0.03	0.02	0.01	0.01
		MuC	74.66	7.58	15.43	1.99	0.19	0.06	0.06	0.03	0	0	0
**B. List of abbreviations (Abv.)**													
Word/phrase			Abv.	Word/phrase				Abv.	Word/phrase				Abv.
Monochrome			Mo	Color appearance				E	Tetragonal phase				t-
Multilayer			Mu	Translucency parameter				TP	Monoclinic phase				m-
Regular sintering time			Hr	Contrast ratio				CR	Cubic phase				c-
Short sintering time			Hs	Opalescence parameter				OP	Black background				B
Ultrashort sintering time			Hu	Color appearance difference				Δ *E*_diff_	White background				W
Extremely short sintering time			He	Lightness				L*	Incisal region				I
Regular sintering temperature			Tr	Red-green				a*	Middle region				M
Decreasing sintering temperature			Td	Yellow-blue				b*	Cervical region				C
Increasing sintering temperature			Ti	Perception threshold				PT	Acceptable threshold				AT

### Determination Color Parameters

Color spectroscopy (ColorQuest XE, Hunter, Reston, VA, United States) was utilized to quantify the optical parameters of Mo 5Y-PSZ and at the incisal (I), middle (M), and cervical (C) regions of Mu 5Y-PSZ specimens. A D65 light with 100% ultraviolet (UV) at 380- to 780-nm wavelength, illuminated through an aperture of 4 mm in diameter, with a 10% observation angle was used to determine the color parameters in the Commission Internationale de l'Eclairage ( CIEL*a*b*) system. The standard white tile ( L* = 96.7, a* = 0.1, b* = 0.2) was utilized for machine calibration before experimentation. A transparent jig was equipped to the specimen for accurate positioning of the measuring location. The values L* , a* , and b* were then computed for color appearance ( *E _w_* ), color appearance difference ( *∆E*_diff_ ), TP, CR, and OP. The *E _w_* was achieved from the lightness ( L* _W_ ), the red-green ( a* _W_ ), and the yellow-blue ( b* _W_ ) coordinates of the specimens on a white background (w) as equation (2) . The color appearances for both Mo and Mu that sintered at regular sintering time (Hr, 135 minutes) and temperature (Tr, 1500°C) were used as a reference. The *∆E*_diff_ was achieved through the difference of L* _W_ , a* _W_ , b* _W_ coordinates of the specimens between the varied (v) and regular (r) sintering parameters according to equation (3) . 15 18 22 29





The TP was computed from the difference between color determinants upon black [(B), L* = 10.4, a* = 0.4, b* = 0.6)] and white [(W), L* = 96.7, a* = 0.1, b* = 0.2)] backgrounds, according to equation (4) . 15 29



The CR was computed using equations (5) and (6) . 15 29 The CR ranged from 0 (transparent) to 1 (absolutely opaque) and was computed from the luminance ( *Y* ) belonging to tristimulus color space/XYZ as determined upon a black background ( *Y _B_* ) and white background ( *Y _w_* ), while *Y _n_* is equal to 100.





The OP was computed according to equation (7) . 15 29



### Evaluation the Microstructure

The samples in every group were simply randomized for microscopic surface examination. The sample was cleansed in purified water, followed by acetone, and then coated with Au-Pd in a sputtering machine (Emitech K500X, Ashford, UK) at 10-mA current, under 130 mTorr vacua, for 3 minutes. The surface characteristics and crystalline structure were evaluated with a scanning electron microscope (SEM; Hitachi, Osaka, Japan) and energy dispersive spectroscopy (EDS; Oxford, Oxfordshire, UK).

### Evaluation the Crystalline Structure

The crystal structure of zirconia samples was evaluated in an X-ray diffractometer (XRD, PANalytical, Empyrean, Almelo, The Netherlands) with Cu-k-α ray scanning from a diffraction angle (2θ) of 25 to 55 degrees for a 2-second interval with a 0.02 step size. The crystalline phase was evaluated by crossing with the Powder Diffraction File (PDF) from the Joint Committee of Powder Diffraction Standard (JCPDS). The peaks found from the PDF-JCPDC nos. 42–1164, 49–1642, and 37–1484 corresponding to the c, t, and m phases were analyzed. The relative intensities of peaks for the m phase ( *I _m_* ), t phase ( *I _t_* ), and c phase ( *I _c_* ) were analyzed by the X'Pert Plus software (Philips, Almelo, The Netherlands). The calculation was performed upon matching a pseudo-Voigt distribution to the courtesy peak and considering the area beneath the curve. Considering the influence of yttria on the lattice parameters, the corrected factor of 1.311 was used to calibrate the nonlinear curve of assimilated intensity ratios against volume fraction. The Garvie–Nicholson formula was applied for calculating the proportion of m phase ( *X _m_* ), t phase ( *X _t_* ), and c phase ( *X _c_* ) as equations 8 , 9 , 10 , respectively. 5







### Statistical Analysis

An analysis of variance (ANOVA) and post hoc Bonferroni multiple comparisons were clarified for the significant effect of altering sintered temperature and time on color parameters of Mo and Mu 5Y-PSZ using statistics software (SPSS V.20, SPSS Inc, Chicago, IL, United States), and justified for statistical significance at 95% confidence interval. Microstructures including grain size and crystalline composition were descriptively determined.

## Results

**Fig. 1:**
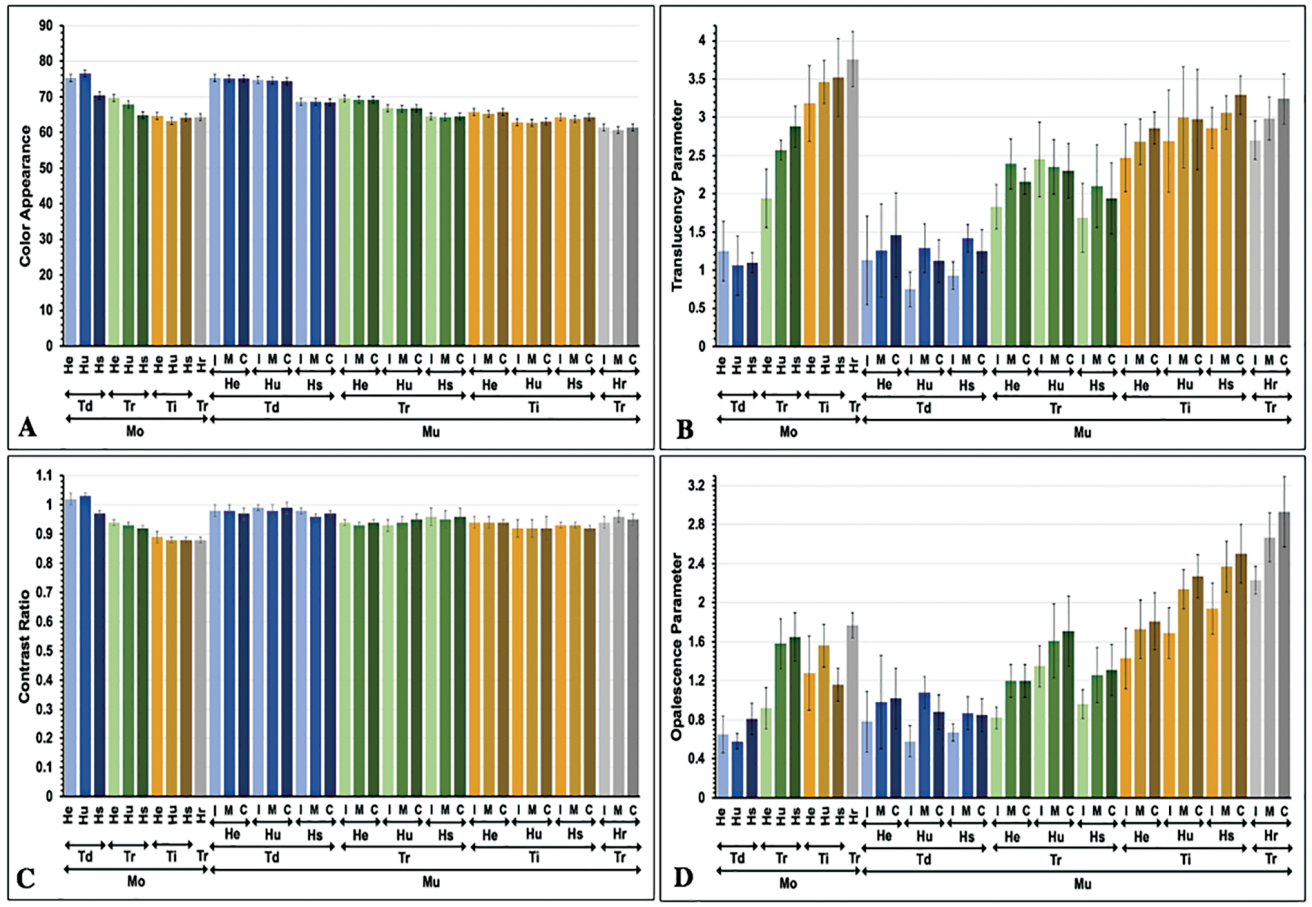
( **A** ) Color appearance, ( **B** ) translucency parameter, ( **C** ) contrast ratio, and ( **D** ) opalescent parameter of monochrome (Mo), and multilayer (Mu) 5Y-PSZ at cervical (C), middle (M), and incisal (I) regions upon decreasing (Td), regular (Tr), and increasing (Ti) sintering temperature with regular (Hr), short (Hs), ultrashort (Hu), and extremely short (He) sintering time.

**Fig. 2:**
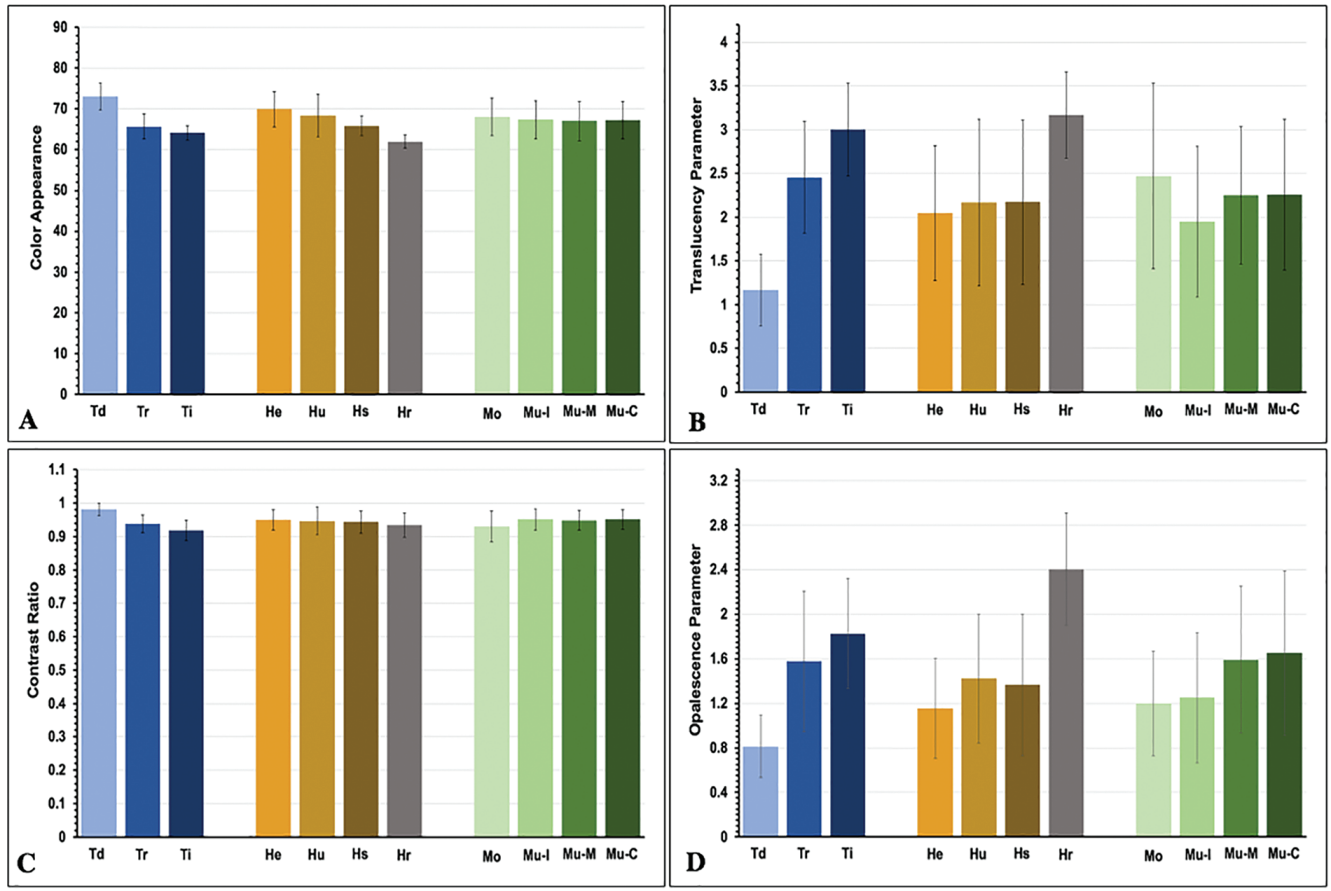
( **A** ) Color appearance, ( **B** ) translucency parameter, ( **C** ) contrast ratio, and ( **D** ) opalescent parameter upon the effect of sintering temperature (decreasing [Td], regular [Tr], and increasing [Ti]), sintering time (regular [Hr], short [Hs], ultrashort [Hu], and extremely short [He]), and different type of 5Y-PSZ (monochrome [Mo], and multilayer [Mu] 5Y-PSZ at cervical [C], middle [M], and incisal [I] regions).

The mean and standard deviation (SD) of color parameters ( *E _W_* , TP, CR, OP, and Δ *E*_diff_ ) of Mo and Mu 5Y-PSZ are presented in Table 2 and Fig. 1 . ANOVA signified that the color parameters, including Δ *E _W_* , TP, CR, OP, and Δ *E*_diff_ , were significantly influenced by zirconia type, sintered time, sintering temperature, and their interactions ( *p* < 0.05), except only the interaction between the type of zirconia and sintered time for Δ *E*_diff_ ( *p* > 0.05) as presented in Table 3 . Post hoc Bonferroni multiple comparisons indicated that types of zirconia presented a statistically significant effect on the *E _W_* , TP, CR, OP, and Δ *E*_diff_ ( *p* < 0.05), except for groups of Multilayer at Incisal region (Mu-I)/Multilayer at Middle region (Mu-M)/Multilayer at Cervical region (Mu-C) in *E _W_* , Mu-M/Mu-C in TP, Mu-I/Mu-M/Mu-C in CR, Mo/Mu-I, Mu-M/Mu-C in OP, and Mu-I/Mu-C in Δ *E*_diff_ ( Table 4 and Fig. 2 ). The study indicated that translucency of Mo zirconia was significantly higher than that of Mu zirconia for all regions ( *p* < 0.05). There was comparable translucency of the M and C regions of Mu zirconia ( *p* > 0.05), and both had a significantly higher translucence than the I region ( *p* < 0.05). The study indicated significantly less contrast of Mo than Mu for all regions ( *p* < 0.05), but comparable contrast of the I, M, and C regions of Mu zirconia ( *p* > 0.05). The opalescence was significantly higher for Mu-C and Mu-M than Mu-I and Mo ( *p* < 0.05); however, a comparable opalescence between Mu-I and Mo was indicated ( *p* > 0.05).

**Table 2:** Mean, standard deviation (SD) of color appearance (E _W_ ), translucency parameter (TP), contrast ratio (CR), opalescent parameter (OP), color appearance difference (Δ *E*_diff_ ), grain size distribution (%), and relative cubic (c), tetragonal (t), and monoclinic (m) phase content (wt%) of monochrome (Mo), and multilayer (Mu) 5Y-PSZ at cervical (C), middle (M), and incisal (I) regions upon decreasing (Td), regular (Tr), and increasing (Ti) sintering temperature with regular (Hr), short (Hs), ultrashort (Hu), and extremely short (He) sintering time

Group			* E _W_* *(mean ± SD)*	TP (mean ± SD)	CR (mean ± SD)	OP (mean ± SD)	∆ *E*_diff_ (mean ± SD)	Grain size distribution			Relative phase		
								Small	Medium	Large	c	t	m
Mo	Td	He	75.22 ± 1.31	1.25 ± 0.39	0.99 ± 0.01	0.65 ± 0.19	12.11 ± 1.15	90	10	0	46.7	52.2	1.2
Mo	Td	Hu	76.51 ± 0.88	1.06 ± 0.39	1.00 ± 0.01	0.59 ± 0.08	13.14 ± 1.52	91.67	8.33	0	44.5	53.3	1.2
Mo	Td	Hs	70.31 ± 0.50	1.1 ± 0.13	0.98 ± 0.01	0.81 ± 0.16	6.87 ± 0.49	88.89	11.11	0	43	54.7	1.3
Mo	Tr	He	69.65 ± 1.30	1.94 ± 0.38	0.94 ± 0.01	0.92 ± 0.21	6.08 ± 0.63	80.49	19.51	0	47.9	50	2.1
Mo	Tr	Hu	67.79 ± 0.81	2.57 ± 0.13	0.93 ± 0.01	1.58 ± 0.26	4.54 ± 0.74	64.10	33.33	2.56	46.7	50.5	2.9
Mo	Tr	Hs	64.85 ± 0.68	2.88 ± 0.27	0.92 ± 0.01	1.65 ± 0.25	1.67 ± 0.53	58.06	35.48	6.45	55.5	43.1	1.5
Mo	Ti	He	64.70 ± 1.49	3.18 ± 0.50	0.89 ± 0.02	1.28 ± 0.38	1.93 ± 0.85	17.65	64.71	17.65	55.4	42	2.5
Mo	Ti	Hu	63.26 ± 0.48	3.46 ± 0.28	0.89 ± 0.01	1.56 ± 0.22	1.84 ± 0.68	7.14	57.14	35.71	52.1	46.3	1.6
Mo	Ti	Hs	64.17 ± 0.57	3.52 ± 0.51	0.89 ± 0.02	1.16 ± 0.17	0.85 ± 0.48	0	38.46	61.54	54.1	44	1.9
Mo	Tr	Hr	64.28 ± 0.81	3.76 ± 0.36	0.88 ± 0.01	1.77 ± 0.13	0	0	33.33	66.67	58.6	39.5	2
Mu-I	Td	He	75.31 ± 2.43	1.13 ± 0.58	0.98 ± 0.02	0.78 ± 0.31	14.33 ± 2.61	88.46	11.54	0	46.4	52	1.6
Mu-M	Td	He	75.12 ± 3.08	1.26 ± 0.61	0.98 ± 0.02	0.98 ± 0.48	14.75 ± 3.18	86.84	13.16	0			
Mu-C	Td	He	75.02 ± 3.05	1.46 ± 0.55	0.97 ± 0.02	1.02 ± 0.31	13.73 ± 2.89	84.09	15.91	0			
Mu-I	Td	Hu	74.73 ± 0.90	0.75 ± 0.23	0.99 ± 0.01	0.58 ± 0.16	13.63 ± 1.47	100	0	0	46.5	52	1.5
Mu-M	Td	Hu	74.52 ± 0.84	1.29 ± 0.32	0.98 ± 0.02	1.08 ± 0.16	14.10 ± 1.40	85.71	14.29	0			
Mu-C	Td	Hu	74.28 ± 0.82	1.12 ± 0.28	0.99 ± 0.02	0.88 ± 0.18	13.01 ± 1.33	88.89	11.11	0			
Mu-I	Td	Hs	68.62 ± 0.54	0.93 ± 0.18	0.98 ± 0.01	0.67 ± 0.09	7.46 ± 0.66	90.91	9.09	0	51.1	48.7	0.3
Mu-M	Td	Hs	68.58 ± 0.64	1.42 ± 0.18	0.96 ± 0.01	0.87 ± 0.17	8.00 ± 0.79	85.37	14.63	0			
Mu-C	Td	Hs	68.39 ± 0.52	1.25 ± 0.28	0.97 ± 0.01	0.85 ± 0.17	7.02 ± 0.66	86.67	13.33	0			
Mu-I	Tr	He	69.47 ± 0.83	1.83 ± 0.29	0.94 ± 0.01	0.82 ± 0.11	8.26 ± 0.92	82.35	17.65	0	50.5	48.1	1.4
Mu-M	Tr	He	69.21 ± 0.77	2.39 ± 0.33	0.93 ± 0.01	1.20 ± 0.17	8.68 ± 0.84	65.79	31.58	2.63			
Mu-C	Tr	He	69.19 ± 0.66	2.16 ± 0.17	0.94 ± 0.01	1.20 ± 0.17	7.82 ± 0.82	78.95	21.05	0			
Mu-I	Tr	Hu	66.87 ± 0.90	2.45 ± 0.49	0.93 ± 0.02	1.35 ± 0.21	5.73 ± 1.17	35.29	58.82	5.88	50.1	48.4	1.4
Mu-M	Tr	Hu	66.64 ± 0.89	2.35 ± 0.36	0.94 ± 0.02	1.61 ± 0.38	6.28 ± 1.22	70.27	27.03	2.70			
Mu-C	Tr	Hu	66.77 ± 0.73	2.30 ± 0.36	0.95 ± 0.02	1.71 ± 0.36	5.41 ± 1.01	73.53	23.53	2.94			
Mu-I	Tr	Hs	64.42 ± 1.16	1.69 ± 0.45	0.96 ± 0.02	0.96 ± 0.15	3.18 ± 1.42	65.38	34.62	0	47.3	50.5	2.1
Mu-M	Tr	Hs	64.28 ± 1.19	2.1 ± 0.54	0.94 ± 0.03	1.26 ± 0.28	3.70 ± 1.37	47.62	52.38	0			
Mu-C	Tr	Hs	64.41 ± 1.04	1.94 ± 0.47	0.96 ± 0.03	1.31 ± 0.26	3.05 ± 1.28	71.43	28.57	0			
Mu-I	Ti	He	65.76 ± 1.13	2.47 ± 0.44	0.94 ± 0.02	1.69 ± 0.26	4.49 ± 1.47	35.29	58.82	5.88	51.4	46.3	2.2
Mu-M	Ti	He	65.23 ± 0.88	2.68 ± 0.30	0.94 ± 0.02	2.14 ± 0.20	4.57 ± 0.99	57.14	35.71	7.14			
Mu-C	Ti	He	65.74 ± 0.74	2.86 ± 0.21	0.94 ± 0.01	2.27 ± 0.22	4.35 ± 0.89	38.46	53.85	7.69			
Mu-I	Ti	Hu	62.79 ± 2.33	2.69 ± 0.67	0.92 ± 0.03	1.43 ± 0.31	2.09 ± 1.56	55.56	38.89	5.56	53.5	44.5	2
Mu-M	Ti	Hu	62.57 ± 2.49	3.00 ± 0.66	0.92 ± 0.03	1.73 ± 0.30	2.53 ± 1.61	38.10	42.86	19.05			
Mu-C	Ti	Hu	63.03 ± 2.43	2.97 ± 0.66	0.92 ± 0.04	1.81 ± 0.29	2.78 ± 1.42	31.82	54.55	13.64			
Mu-I	Ti	Hs	64.34 ± 0.59	2.86 ± 0.27	0.93 ± 0.01	1.94 ± 0.26	3.02 ± 1.01	35.71	57.14	7.14	54.6	43.1	2.3
Mu-M	Ti	Hs	63.66 ± 0.71	3.06 ± 0.22	0.93 ± 0.01	2.37 ± 0.26	2.98 ± 1.15	12.5	62.5	25.00			
Mu-C	Ti	Hs	64.26 ± 0.60	3.29 ± 0.25	0.92 ± 0.01	2.50 ± 0.30	2.91 ± 0.97	7.14	57.14	35.71			
Mu-I	Tr	Hr	61.34 ± 0.87	2.70 ± 0.25	0.94 ± 0.02	2.23 ± 0.14	0	40.00	53.33	6.67	49.6	48.5	1.9
Mu-M	Tr	Hr	60.71 ± 0.88	2.98 ± 0.28	0.96 ± 0.02	2.67 ± 0.25	0	23.53	52.94	23.53			
Mu-C	Tr	Hr	61.42 ± 0.75	3.24 ± 0.33	0.95 ± 0.02	2.93 ± 0.36	0	8.33	58.33	33.33			

**Table 3:** Two-way analysis of variance (ANOVA) of (A) color appearance, (B) translucency parameter, (C) contrast ratio, (D) opalescent parameter, and (E) color appearance difference of monochrome and multilayer 5Y-PSZ upon different sintering temperature and sintering time

**A. ANOVA of color appearance of 5Y-PSZ upon different sintering protocols**					
**Source**	**SS**	**df**	**MS**	***F***	***p*** **-value**
Corrected model	12,131.263	39	311.058	182.391	0.001
Material	117.141	3	39.047	22.896	0.001
Time	2,678.885	3	892.962	523.594	0.001
Temperature	7,485.681	2	3,742.841	2,194.641	0.001
Material × time	85.947	9	9.350	5.600	0.001
Material × temperature	38.096	6	6.349	3.723	0.001
Time × temperature	852.840	4	213.210	125.017	0.001
Material × time × temperature	9.125	12	0.760	0.446	0.001
Corrected total	13,086.313	599			
**B. ANOVA of translucency parameter of 5Y-PSZ upon different sintering protocols**					
Corrected model	414.991	39	10.641	68.830	0.001
Material	22.116	3	7.372	47.687	0.001
Time	42.629	3	14.210	91.916	0.001
Temperature	305.027	2	152.514	986.539	0.001
Material × time	5.501	9	0.611	3.954	0.001
Material × temperature	6.552	6	1.092	7.063	0.001
Time × temperature	8.152	4	2.038	13.183	0.001
Material × time × temperature	8.457	12	0.705	4.559	0.001
Corrected total	501.564	599			
**C. ANOVA of contrast ratio of 5Y-PSZ upon different sintering protocols**					
Corrected model	0.542	39	0.014	34.315	0.001
Material	0.058	3	0.019	47.505	0.001
Time	0.005	3	0.002	4.513	0.001
Temperature	0.367	2	0.183	452.399	0.001
Material × time	0.037	9	0.004	10.016	0.001
Material × temperature	0.049	6	0.008	20.102	0.001
Time × temperature	0.018	4	0.004	10.915	0.001
Material × time × temperature	0.010	12	0.001	2.057	0.018
Corrected total	0.769	599			
**D. ANOVA of opalescence parameter of 5Y-PSZ upon different sintering protocols**					
Corrected model	218.446	39	5.601	87.757	0.001
Material	29.466	3	9.822	153.885	0.001
Time	56.123	3	18.708	293.104	0.001
Temperature	92.315	2	46.158	723.179	0.001
Material × time	9.727	9	1.081	16.934	0.001
Material × temperature	11.060	6	1.843	28.880	0.001
Time × temperature	11.414	4	2.853	44.707	0.001
Material × time × temperature	8.199	12	0.638	10.704	0.001
Corrected total	254.189	599			
**E. ANOVA of color appearance difference of 5Y-PSZ upon different sintering protocols**					
Corrected model	9,830.350	35	280.867	159.782	0.001
Material	248.595	3	82.865	47.141	0.001
Time	1,499.543	2	749.771	426.537	0.001
Temperature	7,161.425	2	3,580.713	2,037.030	0.001
Material × time	1.418	6	0.236	0.134	0.992
Material × temperature	22.623	6	3.770	2.145	0.047
Time × temperature	843.601	4	210.900	119.979	0.001
Material × time × temperature	53.145	12	4.425	2.519	0.003
Corrected total	10,716.287	539			

Abbreviations:5Y-PSZ, 5 mol% yttria-partially stabilized zirconia; df, degree of freedom; F, *F* -ratio; MS, mean square; SS, sum of squares.

**Table 4:** Post hoc Bonferroni multiple comparisons of (A) color appearance, (B) translucency parameter, (C) contrast ratio, (D) opalescent parameter, and (E) color appearance difference of monochrome (Mo) and multilayer (Mu) 5Y-PSZ at cervical (C), middle (M), and incisal (I) regions upon decreasing (Td), regular (Tr), and increasing (Ti) sintering temperature with regular (Hr), short (Hs), ultrashort (Hu), and extremely short (He) sintering time

**(A) Post hoc of color appearance as a function of material, sintering time, and sintering temperature**													
**Material**	**Mo**	**Mu-I**	**Mu-M**	**Mu-C**	**Time**	**He**	**Hu**	**Hs**	**Hr**	**Temperature**	**Td**	**Tr**	**Ti**
Mo	1	.001	.001	.001	He	1	.001	.001	.001	Td	1	.001	.001
Mu-I		1	.227	1	Hu		1	.001	.001	Tr		1	.001
Mu-M			1	1	Hs			1	.001	Ti			1
Mu-C				1	Hr				1				
**(B) Post hoc of translucency parameter as a function of material, sintering time, and sintering temperature**													
**Material**	**Mo**	**Mu-I**	**Mu-M**	**Mu-C**	**Time**	**He**	**Hu**	**Hs**	**Hr**	**Temperature**	**Td**	**Tr**	**Ti**
Mo	1	.001	.001	.001	He	1	.031	.020	.001	Td	1	.001	.001
Mu-I		1	.001	.001	Hu		1	1	.001	Tr		1	.001
Mu-M			1	1	Hs			1	.001	Ti			1
Mu-C				1	Hr				1				
**(C) Post hoc of contrast ratio as a function of material, sintering time, and sintering temperature**													
**Material**	**Mo**	**Mu-I**	**Mu-M**	**Mu-C**	**Time**	**He**	**Hu**	**Hs**	**Hr**	**Temperature**	**Td**	**Tr**	**Ti**
Mo	1	.001	.001	.001	He	1	.660	.016	.001	Td	1	.001	.001
Mu-I		1	1	1	Hu		1	.943	.001	Tr		1	.001
Mu-M			1	1	Hs			1	.006	Ti			1
Mu-C				1	Hr				1				
**(D) Post hoc of opalescence parameter as a function of material, sintering time, and sintering temperature**													
**Material**	**Mo**	**Mu-I**	**Mu-M**	**Mu-C**	**Time**	**He**	**Hu**	**Hs**	**Hr**	**Temperature**	**Td**	**Tr**	**Ti**
Mo	1	.490	.001	.001	He	1	.018	.001	.001	Td	1	.001	.001
Mu-I		1	.001	.001	Hu		1	.973	.001	Tr		1	.001
Mu-M			1	.299	Hs			1	.001	Ti			1
Mu-C				1	Hr				1				
**(E) Post hoc of color appearance difference as a function of material, sintering time, and sintering temperature**													
**Material**	**Mo**	**Mu-I**	**Mu-M**	**Mu-C**	**Time**	**He**	**Hu**	**Hs**		**Temperature**	**Td**	**Tr**	**Ti**
Mo	1	.001	.001	.001	He	1	.027	.001		Td	1	.001	.001
Mu-I		1	.024	.196	Hu		1	.001		Tr		1	.001
Mu-M			1	.001	Hs			1		Ti			1
Mu-C				1									

Post hoc Bonferroni multiple comparisons indicated that varying sintering time had a significant effect on *E _W_* , TP, CR, OP, and Δ *E*_diff_ ( *p* < 0.05), except for groups of He/Hu and Hu/Hs in CR and Hu/Hs in OP ( Table 4 and Fig. 2 ). Sintering zirconia with a shorten sintered time caused significantly less translucency than sintering at a Hr ( *p* < 0.05) for both Mo and Mu 5Y-PSZ. Significantly increasing contrast was indicated upon reducing sintering time compared to Hr ( *p* < 0.05). However, there was comparable contrast between Hs and Hu ( *p* > 0.05) and between Hu and He ( *p* > 0.05). Significantly decreasing in opalescence was indicated upon reducing sintering time compared to Hr ( *p* < 0.05); however, comparable contrast between Hs and Hu was indicated ( *p* > 0.05).

Altering the sintering temperature had a significant influence on the *E _W_* , TP, CR, OP, and Δ *E*_diff_ of color parameters ( *p* < 0.05; Table 4 and Fig. 2 ). The study revealed that the process of sintering 5Y-PSZ at Ti produced a significantly greater translucency and more opalescence, but less contrast and color appearance than sintering at Tr ( *p* < 0.05) for both Mo and Mu zirconia. In contrast, sintering 5Y-PSZ at Td produced significant reduction in translucency and less opalescence, but higher contrast and color appearance than sintering at Tr ( *p* < 0.05) for both Mo and Mu zirconia.

**Fig. 3:**
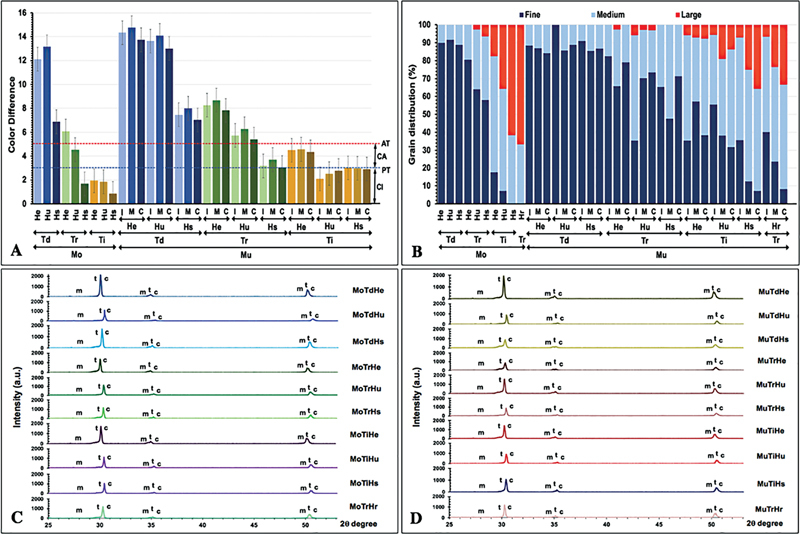
( **A** ) Color appearance difference, ( **B** ) grain distribution, ( **C** ) and relative phase content (t, m, and c phase) of monochrome (Mo) and ( **D** ) multilayer (Mu) 5Y-PSZ at cervical (C), middle (M), and incisal (I) region upon decreasing (Td), regular (Tr), and increasing (Ti) sintering temperature with regular (Hr), short (Hs), ultrashort (Hu), and extremely short (He) sintering time.

Furthermore, the study indicated that the color appearance of zirconia revealed a significant impact from varying sintering temperature and sintering time for both Mo and Mu zirconia ( *p* < 0.05). The color appearance difference (Δ *E*_diff_ ) of zirconia upon varying sintering temperature and time compared to regular sintered temperature and time indicated a significant difference among groups ( *p* < 0.05; Tables 3 and 4 and Fig. 3A ). The study indicated that sintering 5Y-PSZ at Ti resulted in a significantly less color difference than sintering at Tr ( *p* < 0.05), while sintering 5Y-PSZ at Td produced a significantly higher color difference than sintering at Tr ( *p* < 0.05) for both Mo and Mu zirconia. The study indicated that sintering 5Y-PSZ at short sintering time (either He, Hu, or Hs) resulted in a significantly higher color difference than sintering at Hr ( *p* < 0.05) for both Mo and Mu zirconia. Sintering Mo at Ti, sintered at Hs, Hu, or He, revealed Δ *E*_diff_ under the perception threshold (PT). Sintering Mo at Tr with Hs also revealed Δ *E*_diff_ under the perception. However, sintering Mo at Tr with Hu revealed Δ *E*_diff_ under the acceptable threshold (AT). However, sintering Mo at Td, sintered at Hs, Hu, or He, and sintering Mo at Tr with He revealed Δ *E*_diff_ above the AT ( Fig. 3A ). Sintering Mu at Ti, sintered at Hs, or Hu revealed Δ *E*_diff_ under the PT, but sintering at Ti with He revealed Δ *E*_diff_ under the AT. Sintering Mu at Tr with HS revealed Δ *E*_diff_ under the AT. On the contrary, sintering Mo at Td, sintered at Hs, Hu, or He, as well as sintering Mo at Tr with Hu or He, revealed Δ *E*_diff_ above the AT ( Fig. 3A ). The study indicated sintering Mo at Tr, reducing the sintered time to Hs or Hu, can be performed and still obtain clinically acceptable color appearance. While sintering Mu at Tr, reducing sintered the time to Hs, can be performed and still obtain a clinically acceptable color appearance. The study also indicated that sintering Mo or Mu at Td in conjunction with reducing sintering time to Hs, Hu, or He, as well as sintering at Tr with He, resulted in a clinically unacceptable appearance. The study suggested that the sintering process by reducing the sintering time of Hs, Hu or He could be securely performed with a Ti to ensure a clinically acceptable color appearance for both Mo and Mu 5Y-PSZ.

**Fig. 4:**
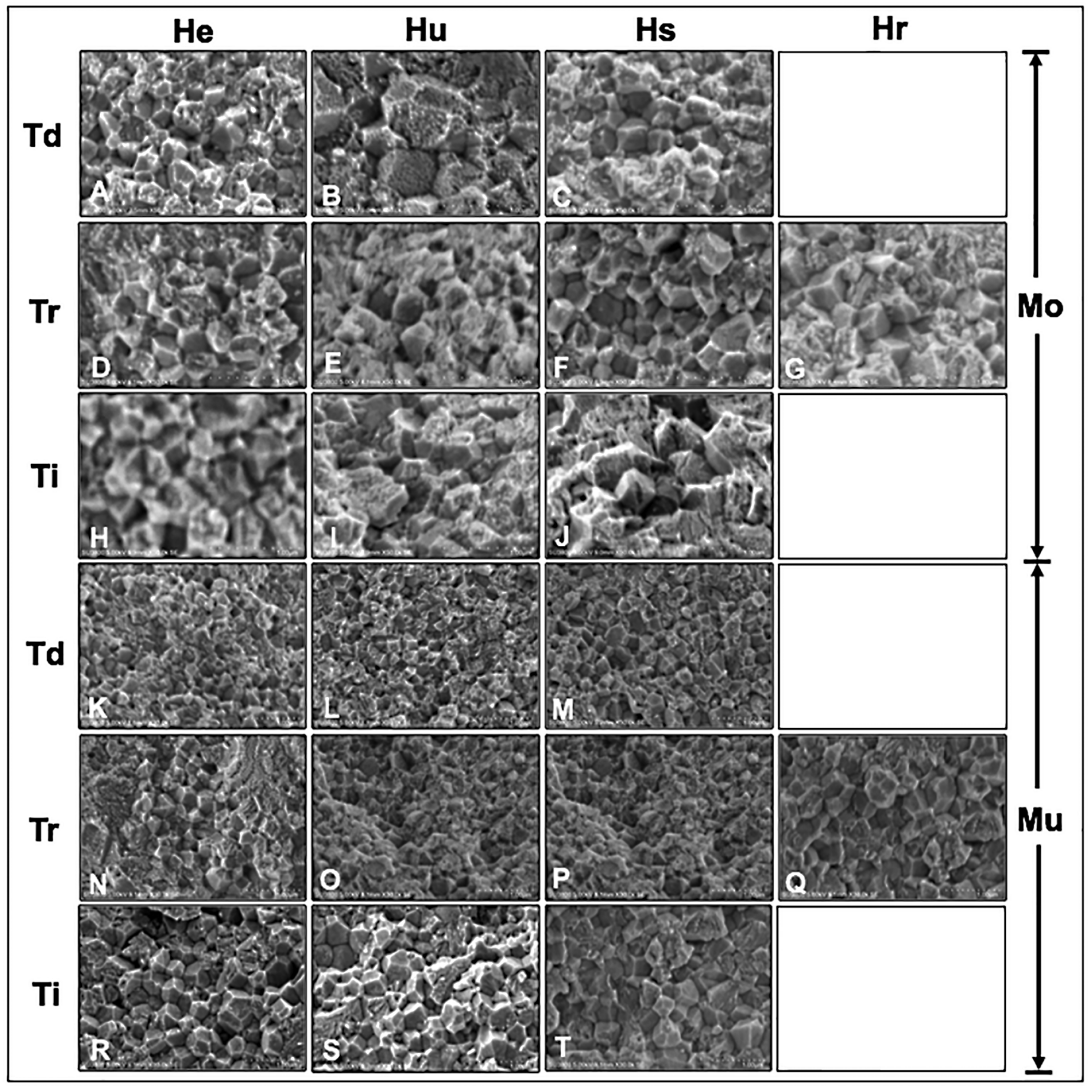
Scanning electron microscope photomicrographs of the crystalline structure indicated grain size and grain distribution at ×30K magnification of ( **A–J** ) monochrome (Mo) and ( **K–T** ) multilayer (Mu) 5Y-PSZ upon ( **A–C, K–M** ) decreasing (Td), ( **D–G, N–Q** ) regular (Tr), and ( **H–J, R–T** ) increasing (Ti) sintering temperature with ( **G, Q** ) regular (Hr), ( **C, F, J, M, P, T** ) short (Hs), ( **B, E, I, L, O, S** ) ultrashort (Hu), and ( **A, D, H, K, N, R** ) extremely short (He) sintering time.

The SEM micrographs of Mo and Mu 5Y-PSZ indicated the different porosity for all groups and appeared in Fig. 4 at ×30K magnification. The microscopic observations denoted the tiniest grain was approximately 0.3 µm and the biggest grain was nearly 2.1 µm. The grain size was classified into three categories: large (1.5–2.1 µm), medium (0.91–1.5 µm), and small (0.3–0.9 µm). The different percentages of grain size distribution were denoted among groups ( Table 2 and Fig. 3B ). The Mo 5Y-PSZ that was sintered at Tr with Hr mainly comprised large grains and minimal amount of medium grains. The Mu 5Y-PSZ that was sintered at Tr with Hr principally comprised medium grains and less amount of large grains. The alterations in sintered parameters led to the different composition of grain sizes ( Fig. 3B and Fig. 4 ). The SEM micrographs denoted the enlargement in grain size and grain growth upon raising the sintering temperature. Thus, there is a correlation between the reduction in grain size and the decreasing sintering temperature. Sintering at Ti resulted in a greater grain size than sintering at Tr or Td. A decrease in grain size was observed when there was sintering at a shortened sintering time. On the other hand, prolonged sintering time exhibited a significant amount of greater grain.

The XRD showed the peak position equivalent with the c, t, and m phases for ZrO _2_ . The intensities in weight percentage (wt%) differed according to zirconia type, sintering temperature, and time. However, all groups indicated the major crystalline structure of c and t phases with a minor amount of m phase ( Table 2 and Fig. 3C, D ). The peak intensity indicated the amount of crystalline structure. The principal peak for t phase was discovered at 2θ of 30.28 degrees that correlated with the 101-crystal configuration. The supplementary peaks for the t phase were observed at the 2θ of 35.271 and 52.28 degrees that correlated with the 2- and 110-crystal configurations. The utmost intensity of peak for c-phases was located at 2θ of 30.28 degrees correlated with the 111-crystalline plane, whereas the others were located at 2θ of 35.29 and 52.31 degrees. The supreme peak intensity for the m phase was discovered at 2θ of 28.48 degrees correlated with the 111-crystal configuration, whereas the minor intense peaks for the m phase were addressed at 2θ of 34.75 and 52.31 degrees that correlated with the 111- and 120-crystal configuration.

## Discussion

This experiment revealed that there are statistically significant alterations of color parameters for both Mo and Mu monolithic zirconia amid changes in sintered time and temperature together with their interactions. Thus, the null hypothesis was rejected for zirconia type, altering sintering time and temperature. Shortening time in the process of ceramic sintering is the preferred criterion for clinicians to consider suitable ceramic sintering process for their restorative reconstruction. The study indicated that upon sintering at Tr, the Mo 5Y-PSZ could be sintered with either Hs or Hu sintered time, while the Mu 5Y-PSZ could be sintered with Hs sintered time, and the resulting color appearance was considered clinically acceptable. Furthermore, upon sintering at Tr with Hs, the color appearance for both Mo and Mu 5Y-PSZ, except for the M region, was below the PT and thus considered a clinically indifferent color appearance. The study indicated that once the sintering process was selected at Tr, shortened sintered time to He for Mo 5Y-PSZ and shortened sintered time to Hu or He for Mu 5Y-PSZ should not be performed, since these led to clinically unacceptable color appearances. The study indicated that decreasing the sintering temperature directed to clinically unacceptable color appearance for Hs, Hu, and He sintered time for both Mo and Mu 5Y-PSZ. This suggested that sintering of either Mo or Mu 5YTZP was not recommended at decreasing sintering temperature to optimize color appearance. Upon sintering at Ti, the Mo 5Y-PSZ could be sintered with either Hs, Hu, or He sintered time, while the Mu 5Y-PSZ could be sintered with either Hs or Hu sintered time, and appeared clinically indifferent. However, the Mu 5Y-PSZ could be sintered at Ti with He sintered time, and still have a clinically acceptable color appearance.

The greater TP value signified the superior translucence material. The current experiment exhibited significantly different TP in each group of materials. The TP value for Mo was the highest, followed by the TP values, in the given order, for Mu-C, Mu-M, and Mu-I. Conversely, Mu-I showed the highest CR value, which was followed, in the descending order, by Mu-M, Mu-C, and Mo. The study indicated that Mo 5Y-PSZ presented more translucence than Mu 5Y-PSZ. It is probably due to the auxiliary pigments incorporated in Mu zirconia producing multiple colors. The pigment particle might create a scattering effect of light and cause decreasing translucence of Mu 5Y-PSZ. Mu 5Y-PSZ exhibited a significant difference in translucency between layers including the difference between the Mu-I and Mu-M regions and between the Mu-I and Mu-C regions. Nevertheless, no significant difference between the Mu-M and Mu-C regions was indicated. The difference in translucency between layers of Mu zirconia might be due to numerous inconsistencies of pigment particles. Even though the data fact sheet from the manufacturer labeled was similar in the major compositions for both Mo and Mu, the study found that the different compositions between layers of Mu zirconia were the colorant additives as indicated by the EDS analysis ( Table 1 ), which probably caused differences in translucence among layers. 6 30 However, no current data indicated the transition of elements owing to varied sintering parameters. The slight difference in composition of Mo 5Y-PSZ upon different sintering protocols probably comes from the dispersion of elements on different fields of EDS and should be further investigated.

This study indicated that modifying the sintering time and temperature disturbed the translucency of 5Y-PSZ. Increasing the sintering temperature resulted in a significant increase in TP, but significantly reduced CR of Mo and Mu 5Y-PSZ. On the contrary, decreasing the sintering temperature resulted in a significantly reduced TP, but significantly increased CR of Mo and Mu 5Y-PSZ. The finding of the present study are supported by other studies that found that sintering zirconia above 1500°C resulted in enlarged grain size and increased translusence. 26 27 The translucency of 5Y-PSZ is affected not only by altering the sintering temperatures but also by varying the sintering times. Shortening the sintering time notably reduced the translucence of Mo and Mu 5Y-PSZ, as indicated by decreasing in TP upon shortened sintering time. Increasing the sintering temperature or extending the sintering time may shrink the pore size and boost condensed zirconia, which promotes better transmission and diminishes the scattering of light, thus exhibiting high translucency. 12 This phenomenon is perhaps related to an isotropic configuration of the crystalline structure, which promotes better transmission and reduces light refraction. The SEM photomicrographs presented with grain growth of zirconia when the sintering temperature and time were increased. Hence, a reduction in pore size in combination with compression of crystal structure was expected to expedite the homogeneousness of the zirconia structure. The number of pores also reduced as the adjacent grains coalesced and formed a larger grain, consequently reducing grain boundaries and lessening scattering of light at the boundaries, thus enhancing translucency. Once the sinter temperature was lowered, there was insufficient energy for diffusion of particles, thus inhibiting grain compaction. The study suggested sintering the zirconia at an increasing sintering temperature to provide a better translucency as well as appropriate strength. 13 31 On the other hand, decreasing the sintering temperature or time resulted in porosity in the microstructure that involves the translucency of zirconia. Inadequate growth of grain for crystalline structure, usually presented with several fine grains, subsequently indicated numerous grain boundaries, induced more scattering on boundaries, and decreased light passing through the grain, thus undoubtedly reducing translucence of 5Y-PSZ, as witnessed in the groups of the shortened sintering time. The study was endorsed by others that reported tinier grains as inadequate grain growth caused a lessened translucence of zirconia. 4 18 22 The results of the study support the notion that enhancing the translucence of zirconia requires amplification of grain. The microstructure analysis using XRD indicated that the crystal structure of 5Y-PSZ comprised mainly c and t phases with minor amounts of m phase in every group. However, the intensity of the c phase may be slightly different among the brands of 5Y-PSZ. This study indicated that varying the sintering parameters influenced the phase concentration. Elevating the sintering temperature and sintered time likely elevated the c phase. Moreover, increasing the sintering temperature possibly activates stress inside the zirconia microstructure and accordingly initiates a t to m phase transformation as supported by other studies. 18 31 An increase in the m phase upon increasing the sintered temperature possibly engendered a higher scattering effect because the difference in refractive index of the majority of the c and t phases and the tiny of m phase. Likewise, extending the sintering time probably generates better consistency of the crystal configuration, which results in superior light transmission. This study strongly confirmed that rising sintering temperature and time resulted in increasing translucency of 5Y-PSZ. Accordingly, increasing the sintering temperature is recommended as it enhances the flexural strength of 5Y-PSZ as supported by other studies. 4 12 30

Opalescence is also an interesting parameter. The restorative material should possess an OP close to that of humans to resemble the appearance of a human tooth. In this study, the OPs for Mo and Mu 5Y-PSZ were 0.59 to 2.93, which was less than that of the human enamel (19.8–27.6). 21 There was a significant difference in the OP among the layers of Mu 5Y-PSZ. The OP trended to increase when the sintering time and temperature were increased. Zirconium oxide and yttrium oxide in the composition of ceramic are affected by the OP. There were reports that the opalescence of ceramics increases with an increase in concentration of a particular oxide, for example, ZrO _2_ , Y _2_ O _3_ , SnO _2_ , and V _2_ O _5_ . 2 Mu-C exhibited the highest OP, followed by Mu-M, Mu-I, and Mo. This is probably related to the coloring pigments, which affected the path of light and resulted in different levels of opalescence.

## Conclusion

The findings of the study can be summarized as follows. Altering the sintering parameters significantly affects the color parameters of both Mo and Mu monolithic 5Y-PSZ. Adjustment of sintering parameters to achieve good color characteristics of zirconia with appropriate processing time for chairside restorative treatment is feasible. Decreasing the sintering time to Hs, Hu, or He for Mo 5Y-PSZ to Hs or Hu for Mu 5Y-PSZ is feasible but the sintering temperature must be increased to achieve the most favorable color appearance, translucence, contrast, and opalescence with a clinically indifferent color appearance. Nevertheless, decreasing the sintering time to He for Mo 5Y-PSZ is possible with the need to compensate for that with increasing the sintering temperature to achieve favorable color parameters with a clinically acceptable color appearance. However, decreasing the sintering temperature for either Mo or Mu 5Y-PSZ is not recommended for sintering at HS, Hu, or He sintering time, as it results in unfavorable color parameters with clinically unacceptable color appearance. Decreasing the sintering temperature and time causes a reduction in grain size, t to m conversion, and ultimately poorer translucence and less opal. This investigation supports the proposition that to achieve better translucence and opal of 5Y-PSZ, the sintering temperature must be increased when the sintering time is shortened. Decreasing the sintering temperature of 5Y-PSZ is not recommended as leads to unfavorable color characteristics with a clinically unacceptable color appearance. Decreasing the sintering time of 5Y-PSZ is possible, but the sintering temperature must be increased to achieve the most promising color parameters with a clinically indifferent color alteration. Hence, to achieve the most auspicious optical characteristics, increasing the sintering temperature is recommended in case the sintering time is shortened to facilitate a chair-side reconstruction with either Mo or Mu 5Y-PSZ.
